# The Michigan Autism Spectrum Questionnaire: A Rating Scale for High-Functioning Autism Spectrum Disorders

**DOI:** 10.1155/2013/708273

**Published:** 2013-12-05

**Authors:** M. Ghaziuddin, K. Welch

**Affiliations:** ^1^Department of Psychiatry, University of Michigan Medical Center, Plymouth Road, Ann Arbor, MI 48109-0277, USA; ^2^Center for Statistical Consultation and Research (CSCAR), University of Michigan Medical Center, Plymouth Road, Ann Arbor, MI 48109-0277, USA

## Abstract

Although the DSM-5 has recently created a single category of autism spectrum disorder (ASD), delineation of its putative subtypes remains clinically useful. For this process, screening instruments should ideally be brief, simple, and easily available. The aim of this study is to describe the validity of one such instrument. We administered the Michigan Autism Spectrum Questionnaire (MASQ), a 10-item questionnaire, to 42 patients with ASD (age range 6–13 years, mean 9.7 years, SD 2.5, one female) and 18 patients with other psychiatric disorders (age range 6–17 years, mean 11.7 years, SD 3.8, 6 females). Responses to each item were scored from 0 to 4 yielding a total score of 30. Patients with intellectual disability were excluded. As a group, patients with ASD scored higher than those with other psychiatric disorders (Chi-square test with 1 df = 16.019, *P* < 0.0001). Within the ASD group, a linear discriminant analysis found that the best cut-off points were 22 or above for Asperger syndrome, 14 to 21 for autism/PDDNOS, and less than 14 for those with other psychiatric disorders. We propose that the MASQ can be used as a brief measure to screen high-functioning ASD from other psychiatric disorders and to identify its possible subtypes.

## 1. Introduction

Autism is a developmental disorder characterized by a distinct pattern of social and communication impairment with rigid ritualistic interests first described by Kanner [1]. It is sometimes divided into high- and low-functioning types depending on the level of intelligence of the affected individual. Almost 40 years after its initial description, it was introduced as infantile autism in the DSM-III [2]. Subsequently, the DSM-III-R [3] introduced the concept of Pervasive Developmental Disorders (PDD) to describe a group of conditions marked by similar deficits of socialization, communication, and imagination, consisting of the main category of autistic disorder and a residual entity of Pervasive Developmental Disorder Not Otherwise Specified (PDDNOS). Reflecting the increasing interest in Asperger Disorder (or Asperger syndrome) [4], the DSM-IV [5] added it to the group of PDD along with Rett's Disorder and Disintegrative Disorder and retained PDDNOS. However, the recently published DSM-5 has combined autistic disorder, Asperger syndrome, and PDDNOS into a single category of autism spectrum disorder (ASD) and eliminated Rett's and Disintegrative Disorders [6]. The decision to merge the three disorders was apparently taken because they could not be easily distinguished from each other. However, differentiating Asperger syndrome from autism has clinical utility, and delineating the subtypes of the autism spectrum has treatment implications [7, 8]. For example, children with Asperger syndrome may respond better to different kinds of communication skills training than those with autism. Likewise, certain kinds of behaviors and symptoms may be more common in some individuals with high-functioning autism and Asperger syndrome than in those with low-functioning autism [9]. Although several rating scales exist for the assessment of autism spectrum disorders, their diagnosis remains expensive and time consuming, especially in those who are high functioning and who suffer from comorbid psychiatric conditions. In this preliminary study, we describe the use of a brief rating scale to screen for the presence of high-functioning autism spectrum disorders in psychiatric settings.

## 2. Method

### 2.1. Participants

The study was conducted at the University of Michigan Child Psychiatry Clinic in Ann Arbor, USA. The sample consisted of forty-two participants with autism spectrum disorders (age range 6–13 years, mean 9.78 years, SD 2.5, one female). Of these, 15 had Asperger syndrome (AS), 5 had autistic disorder (autism), and 22 had PDDNOS based on the DSM IV-TR criteria [5]. Because of the low numbers of patients with autism and because one purpose was to determine if the scale can identify patients with Asperger syndrome, the autism/PDDNOS groups were merged into a single category. Eighteen participants with other psychiatric disorders (mixed group, age range 6–17 years, mean 11.7 years, SD 3.8, 6 females) formed the comparison group. Comorbid psychiatric disorders in the autistic group were ADHD only (*n* = 19), Mood/Anxiety Disorder only (4), ADHD plus Mood/Anxiety Disorder (5), and Specific Language Impairment (SLI) (1). Eight subjects did not have any comorbid disorder. Psychiatric disorders in the mixed group consisted of ADHD only (*n* = 7), ADHD/Conduct Disorder (*n* = 2), Mood/Anxiety Disorder only (*n* = 5), ADHD plus Mood/Anxiety Disorder (*n* = 3), and SLI plus ADHD (*n* = 2). In all, 9 patients had more than one diagnosis. Since the focus of the study was on high-functioning individuals, participants with a full scale IQ below 70 were excluded. In the absence of an IQ, the level of functioning was estimated on clinical grounds.

### 2.2. Materials

For the diagnosis of ASD, each subject was administered an assessment battery consisting of a brief neuropsychological examination; a speech and language assessment; and a detailed psychiatric interview. In addition, caregivers completed the Child Behavior Checklist [10]; the Conner's Parent Rating Scale-Revised Long Form [11]; the Autism Behavior Checklist [12]; and the Social and Communication Questionnaire [13]. All available records were also examined. The Michigan Autism Spectrum Questionnaire (MASQ) was designed on the basis of the clinical characteristics of high-functioning autism and Asperger syndrome described in the literature. The purpose was to construct a scale that would be brief and easy to administer and incorporate questions targeting behaviors suggestive of Asperger syndrome. The aim was to focus on two main areas: quality of social interactions and form/content of communication. For example, questions 2 to 5 were intended to capture the pedantic style of communication said to be typical of this condition [14]. Question 8 attempted to describe the “active but odd” style of social interaction said to be common in Asperger syndrome as opposed to the “aloof and passive” manner typical of high-functioning autism [15]. Question 9 reflected the clinical impression that persons with AS tend to speak fluently by three years of age and sometimes even earlier, while question 10 referred to the fact that in many cases, features of AS become more apparent as the child grows older, usually by 7-8 years of age. Each question had four responses ranging from 0 to 4, yielding a total score of 30. Approval to perform the study was obtained from the Institutional Review Board (IRB).

*Data Analysis*. Data were analyzed to answer two questions. First, can the MASQ differentiate between autism spectrum disorders and other psychiatric disorders? Second, can the MASQ differentiate between Asperger syndrome and other autistic spectrum disorders?

**Figure 1:**
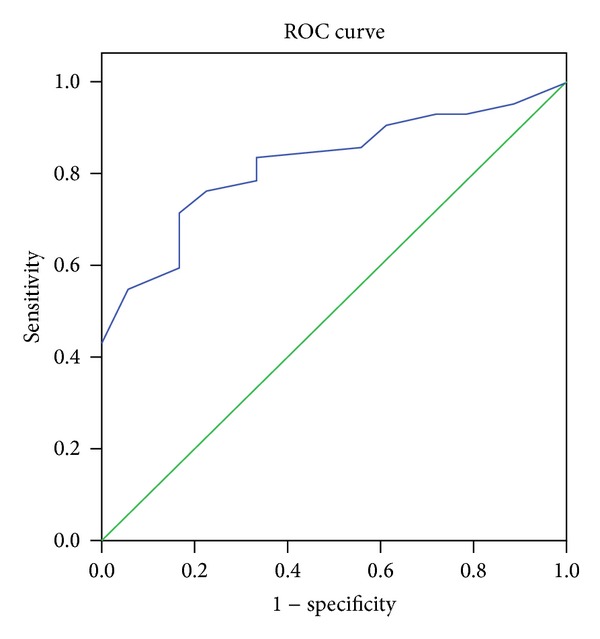
Diagonal segments are produced by ties. Roc Curve for AS, Aut./PDDNOS versus mixed subjects.

To determine if the MASQ can differentiate between ASD and other psychiatric disorders, a binary logistic model on the total MASQ score was used. This model was found to be significant (Likelihood Ratio Chi-square test with 1 df = 16.019, *P* < 0.0001; the Nagelkerke *R*-square was 0.332, and the Area Under Curve was 0.821). A discriminant analysis showed that the total score correctly classified 73.3% of the entire group, 85.7% of the ASD group, and 44.4% of the mixed group as shown in Figure 1.

To find out if the MASQ can differentiate between the various subtypes of ASD, the sample was divided into three groups: 15 with Asperger syndrome, 22 with autism/PDDNOS, and 18 with other psychiatric disorders. Using a multinomial logistic regression, the MASQ scores of the three groups were compared. The overall model was again found to be significant (Likelihood Ratio Chi-square with 2 df = 28.579, *P* < 0.001; Nagelkerke *R*-square was 0.430). Thus, the total score could distinguish between AS and autism/PDDNOS (Wald Chi-square = 8.116, 1 df, *P* = 0.0004) and also between AS and other psychiatric disorders (Wald Chi-square = 15.338, 1 df, *P* < 0.0001).

To ascertain the best cut-off points from the total score that could discriminate between the three groups, we used a linear discriminant analysis. The cut-off points were 22 or above for Asperger syndrome, 14 to 21 for autism/PDDNOS, and less than 14 for the mixed group. Using a discriminant function analysis, 66.7% of patients were correctly classified overall, and 66.7% were correctly classified in each diagnostic group. The leave-one-out cross-validation classification also correctly classified 66.7% of the patients as shown in Table 2.

## 3. Results

Participants with ASD scored higher than those with other psychiatric disorders as shown in Table 1. Within the ASD group, those with Asperger syndrome scored higher than those with autism and PDDNOS.

## 4. Discussion

These findings suggest that the MASQ can screen patients with high-functioning autism spectrum disorders from other psychiatric conditions and to identify patients with possible Asperger syndrome. In this clinic sample, patients with ASD scored higher than those with other psychiatric disorders, and, within the ASD group, those with Asperger syndrome scored the highest. Thus, the highest total scores (>22) predicted Asperger syndrome, the intermediate scores (14 through 21) predicted autism/PDDNOS, and the lowest scores (<14) predicted other psychiatric disorders. Thirteen out of 15 participants (86.7%) with Asperger syndrome scored 20 or higher. On the other hand, the autism/PDDNOS group had a wider range of scores, with 10/27 (26%) scoring 20 or higher and 6/27 (22%) scoring less than 14. One of the 18 participants in the mixed group (5.6%) scored 20, while 12/18 (66.7%) scored less than 14. The fact that 86% of the AS group scored higher than 20 while that autism/PDDNOS group had a much wider spread suggests that in this sample subjects with AS were more homogeneous than those with autism/PDDNOS. This also suggests that within the group of high-functioning autistic spectrum disorders, a subgroup of individuals may be identified based on the *quality* of social interaction and the *style* of communication rather than on the presence/absence of language delay, which is dependent on parental recall. If the central feature of Asperger syndrome is the history of language delay, it cannot be reliably differentiated from high-functioning autism. If, however, the disorder is conceptualized primarily on the *quality* of social deficits, the presence of a *distinct manner of communication*, and the occurrence of focused excessive interests, it may be possible to diagnose a distinct group of affected individuals. As opposed to most rating scales of Asperger syndrome, which rest on the premise that it is a mild form of high-functioning autism, this scale attempts to incorporate the clinical variables of AS that may define its qualitative differences from other types of autism spectrum disorders. While rating scales and structured interviews are not diagnostic on their own and only serve to collect data in a systematic and comprehensive manner, these results suggest that within the ASD spectrum, the scores may index different subtypes. However, before generalizing the findings of the study, it is important to discuss some of its limitations. First, the sample consisted of a relatively small number of participants, mostly males, underscoring the need to replicate the findings in a large group, with adequate numbers of patients with autism, Asperger syndrome, and PDDNOS. Also, the average age of the ASD group was about 10 years. As the symptoms of those with Asperger syndrome and higher functioning autism tend to become more apparent with age, the scale may prove to be equally useful in older adolescents and young adults. Second, as the sample was drawn from an outpatient psychiatric clinic, it will be important to replicate the findings in community samples.

In summary, this pilot study describes the use of a brief rating scale to distinguish between high-functioning autism spectrum disorders (autism and Asperger syndrome) and other psychiatric disorders. The highest total scores (>22) predicted Asperger syndrome while the intermediate scores (14 through 21) predicted autism/PDDNOS. In a psychiatric clinic setting, the MASQ can be used as a simple screening tool to identify children with suspected autism and Asperger syndrome. Future studies should examine its utility in both clinic and community samples.

## Figures and Tables

**Table 1:** MASQ scores of the three groups.

	Asperger syndrome	Autism/PDDNOS	Mixed group
Number	15	27	18
Male : female	15 : 0	26 : 1	12 : 6
Mean age (SD)	9.26 (2.5)	10.07 (2.5)	11.72 (3.8)
Mean MASQ (SD)	21.86 (4.3)	16.96 (4.5)	12.88 (3.9)

**Table 2:** Classification of the sample by the MASQ.

Observed	Predicted			
	AS	Aut/PD NOS	Mixed	Percent correct
AS	10	4	1	66.7%
Aut./PDDNOS	3	19	5	70.4%
Mixed	0	10	8	44.4%
Overall percentage	21.7%	55.0%	23.3%	61.7%

Overall, 61.7% of patients were correctly classified (66.7% of AS patients, 70.4% of Aut./PDD patients, and 44.4% of mixed patients).

## Appendix

### Michigan Autism Spectrum Questionnaire (MASQ)

*Circle the Appropriate Answer for Each Question.*

1. Is he/she able to have a back and forth conversation with you?

**Table:**

Not at all	Just a little	Pretty much	Very much
0	1	2	3

(2) Does he/she have special interests or hobbies that seem to be excessive compared to those of other people of the same age? (Examples: interest in astronomy; roads and bridges; Japanese culture, etc.)

**Table:**

Not at all	Just a little	Pretty much	Very much
0	1	2	3

(3) Does he/she have a tendency to talk excessively on certain topics? (Examples: speaks like a “Little Professor,” pays no attention to the needs of the listener; appears to go on talking even when not necessary, etc.)

**Table:**

Not at all	Just a little	Pretty much	Very much
0	1	2	3

(4) Does he/she offer excessive details about certain topics while speaking? (Examples: gives precise information about animals beginning with the Letter B; offers details about the classification of dinosaurs, etc.)

**Table:**

Not at all	Just a little	Pretty much	Very much
0	1	2	3

(5) Does he/she have an odd manner of speaking? (Examples: uses uncommon or high-flown words; speaks in a formal manner; has an odd intonation, etc.)

**Table:**

Not at all	Just a little	Pretty much	Very much
0	1	2	3

(6) Does he/she make an attempt to interact with others in an inappropriate manner? (Examples: on meeting a person, asks intrusive details about his/her marriage or salary; keeps staring at a stranger just to be “friendly with,” appears over formal and stilted when not expected to do so, etc.)

**Table:**

Not at all	Just a little	Pretty much	Very much
0	1	2	3

(7) Does he/she appear intelligent yet, lacking in common sense?

**Table:**

Not at all	Just a little	Pretty much	Very much
0	1	2	3

(8) Does he/she appear to be physically clumsy? (Examples: bumps into objects; breaks things without realizing it, etc.)

**Table:**

Not at all	Just a little	Pretty much	Very much
0	1	2	3

(9) Did he/she speak at the normal age when growing up? (Example: by 3-4 years of age was able to speak in simple three word sentences, such as, “Give me milk”)

**Table:**

Not at all	Just a little	Pretty much	Very much
0	1	2	3

(10) When did you first suspect that something was different or unusual with your child's behavior?

**Table:**

Before 3 years of age	Between 3 and 4 years	Between 4 and 5 years	After 5 years of age
0	1	2	3

Total Score….
